# Checking behavior in rhesus monkeys is related to anxiety and frontal activity

**DOI:** 10.1038/srep45267

**Published:** 2017-03-28

**Authors:** Marion Bosc, Bernard Bioulac, Nicolas Langbour, Tho Hai Nguyen, Michel Goillandeau, Benjamin Dehay, Pierre Burbaud, Thomas Michelet

**Affiliations:** 1 Université de Bordeaux, Institut des Maladies Neurodégénératives, UMR 5293, F-33000 Bordeaux, France; 2 CNRS, Institut des Maladies Neurodégénératives, UMR 5293, F-33000 Bordeaux, France; 3 CHU de Bordeaux, Service d’explorations fonctionnelles du système nerveux, F-33000 Bordeaux, France; 4 Centre Hospitalier Henri-Laborit, 370, avenue Jacques-Cœur, F-86021, Poitiers, France; 5 CNRS, Institut de Neurosciences Cognitives et Intégratives d’Aquitaine, UMR 5287, F-33000 Bordeaux, France

## Abstract

When facing doubt, humans can go back over a performed action in order to optimize subsequent performance. The present study aimed to establish and characterize physiological doubt and checking behavior in non-human primates (NHP). We trained two rhesus monkeys (*Macaca mulatta*) in a newly designed “Check-or-Go” task that allows the animal to repeatedly check and change the availability of a reward before making the final decision towards obtaining that reward. By manipulating the ambiguity of a visual cue in which the reward status is embedded, we successfully modulated animal certainty and created doubt that led the animals to check. This voluntary checking behavior was further characterized by making EEG recordings and measuring correlated changes in salivary cortisol. Our data show that monkeys have the metacognitive ability to express voluntary checking behavior similar to that observed in humans, which depends on uncertainty monitoring, relates to anxiety and involves brain frontal areas.

Did I really lock my door? We have all experienced this subtle feeling of discomfort after leaving home, sometimes resulting in us returning to check! Checking behavior is essential to maximizing gain and/or minimizing loss in our daily lives and relies on a normal action monitoring process. Its exacerbation is also well known to be a central feature of obsessive-compulsive disorder (OCD), a psychiatric disease that afflicts 2–3% of the population^1^,^2^,^3^. Although the neurobiological bases of uncertainty and doubt have been the subject of considerable study^4^,^5^,^6^,^7^, the lack of fundamental data derived from relevant animal models has contributed to the physiology and physiopathology of checking behavior remaining poorly understood^8^,^9^.

Despite skepticism about the ability of non-human animals to perform cognitive processes such as checking behavior^10^,^11^,^12^, there is increasing evidence for metacognitive capacities in several species^8^,^13^. Monkeys, and more recently rats, have shown the capacity to evaluate their own knowledge or memory^14^ and when doubting their recall ability, to escape from a trial^4^,^5^,^6^,^14^,^15^ or attempt to collect more information^16^,^17^,^18^,^19^,^20^. These behavioral responses resemble those found in humans facing uncertainty^8^,^9^,^15^. Several species have also been tested with information seeking paradigms in either manual or computerized tasks, during which subjects were offered the possibility to seek for more information or a second opportunity to check the sensory evidence before making their decision^16^,^18^,^19^,^20^,^21^,^22^,^23^,^24^,^25^,^26^,^27^,^28^,^29^. Among these cases, baboons^25^ and chiefly rhesus monkeys^16^,^19^,^20^ and apes^18^,^27^,^29^ were able to use these alternative opportunities in order to improve their performance when lacking enough information to answer directly. Moreover, recent studies have emphasized the close homology existing between human and monkey brain structures involved in cognitive tasks^30^,^31^. We therefore hypothesized that rhesus monkeys are likely to express voluntary checking behavior based on their own doubts when facing uncertainty about a previous decision, and then use it as an adaptive function to improve their performance.

Checking can be simply defined as going back over a goal-directed behavior in response to self-doubt^32^. The main aim of laboratory animals in any goal-directed decision-making is to obtain a reward. Based on this assumption, we developed a “Check-or-Go” behavioral task in which a monkey could select and check the availability of a reward that was delivered upon the correct accomplishment of a simple visuo-motor task (Fig. 1a). This Check-or-Go task combined two main sequential steps, a “Selection-step” and a “Confidence-Report-step” (Fig. 1b; Supplementary video 1, 2). During the Selection-step, reward availability was indicated on a touch-screen monitor through a switchable “REward Control” (REC) cue that could be predominantly green (reward status “On” = available) or red (reward status “Off” = unavailable). The monkey could either press the REC-cue itself to switch the status from “Off” to “On” or *vice versa*, or move directly to the second step by pressing the “next-step” arrow (>>). In order to challenge the animal’s degree of certainty and raise doubt about reward availability, we used three levels (low, medium and high) of perceptual ambiguity by modulating the green/red ratio of the REC-cue (Fig. 1c).

We first looked at the influence of perceptual ambiguity on the decision-making process during the Selection-step. The monkey’s performances were modulated by REC-cue ambiguity level. We defined a trial as successful when the selected reward-status was “On” (i.e green REC-cue selected) upon entry into the final evaluation step (Fig. 1b). The percentage of success (calculated as [number of green REC-cue selected trials/total number of executed trials]% for each ambiguity level) decreased as the level of ambiguity increased (main effect REC-cue ambiguity *Χ*^2^_2,*n*=111782_ = 15117.4; *P* < 0.001; Fig. 2a). Thus, success in low ambiguity trials (98.1%) was significantly higher compared with that in medium (85.8%) and high (63.8%) ambiguity trials (*Χ*^2^_1,*n*=71399_ = 3462.8; *P* < 0.001 vs medium; *Χ*^2^_1,*n*=74951_ = 13360.2; *P* < 0.001 vs high ambiguity). The success rate in medium ambiguity trials was also significantly higher compared to high ambiguity trials (*Χ*^2^_1,*n*=77214_ = 4875.2; *P* < 0.001). REC-cue ambiguity also impacted on the monkey’s reaction time (RT), which increased with the level of ambiguity (main effect REC-cue ambiguity *F*_2,75255_ = 770.5; *P* < 0.001; Fig. 2b). Accordingly, the RT (mean ± sem) in low ambiguity trials (329.6 ± 0.4) was significantly shorter compared with the RT in medium (346.7 ± 0.4) and high (348.4 ± 0.4) ambiguity trials (*t*_54168_ = −32.8, *P* < 0.001 vs medium; *t*_49280_ = −34.3, *P* < 0.001 vs high ambiguity) and the RT in medium ambiguity trials was significantly shorter than in high ambiguity trials (*t*_47056_ = −3.0, *P* < 0.01).

The modulation of certainty has previously been reported in humans^15^,^33^,^34^ and several animal species^4^,^5^,^6^,^34^,^35^. This was identified mostly during perceptual decision-making with visual^5^,^6^,^15^,^34^, olfactory^4^ or auditory^15^ discrimination paradigms, but also using a working memory task^17^ or episodic memory testing^33^. As in most of these studies, we reduced the difference between two stimulus input categories (in our case the “On” and “Off” REC-cue) to force the animal to make a difficult perceptual discrimination (green *versus* red, see Fig. 1c for example). The monkey’s behavior during the Selection-step was influenced by the REC-cue ambiguity level, displaying a clear and consistent impact of perceptual ambiguity on the decision-making process relative to reward expectancy^4^,^5^. We observed a decrease in behavioral response accuracy and an increase in RT under the most ambiguous conditions, attesting to the consequences of increased difficulty as previously reported in humans^15^,^33^,^34^ and animals^4^,^5^,^6^,^34^,^35^. These initial results thus confirmed that in using three levels of perceptual ambiguity, we were able to influence and control the monkey’s certainty during the Selection-step procedure.

During the Confidence-Report step, the monkey could decide either to check reward availability by returning to the Selection-step or proceeding to the trial’s final evaluation step by performing a simple (over 99% correct responses) color-matching task (see Supplementary Videos 1, 2). Generating doubt about reward availability increased behavioral RT for color matching, highlighting the prolonged impact of uncertainty on the animal’s confidence and motivation throughout the decision-making process (Fig. 2c). More importantly, raising doubt also led to checking. Indeed, checking rate increased with the level of REC-cue ambiguity (*Χ*^2^_2,*n*=117186_ = 1814.1, *P* < 0.001), with the checking rate (calculated as [number of checked trials/total number of executed trials]% for each ambiguity level) being significantly higher for medium (6.07%) and high (6.47%) than for low (0.59%) ambiguity trials (*Χ*^2^_1,*n*=74000_ = 1640.7, *P* < 0.001 vs medium; *Χ*^2^_1,*n*=77964_ = 1799.4, *P* < 0.001 vs high ambiguity). Checking rate was also significantly higher for high than medium ambiguity trials (*Χ*^2^_1,*n*=82408_ = 5.5, *P* = 0.01). Checking behavior occurred more often during “Off” than “On” trials (Fig. 3a; *Χ*^2^_1,*n*=117186_ = 1134.7, *P* < 0.001) but its occurrence increased with the ambiguity level, independently of the reward status. Overall, monkeys benefited significantly from deciding to check (Fig. 3b): a statistical analysis of checked trials revealed an increase in the overall proportion of successful trials following (79.8%), as compared to preceding (25.7%), checking initiation (*X*^2^_1,*n*=4834_ = 2838.1, *P* < 0.001).

Interestingly, the checking rate as a function of the animal’s performance and ambiguity of the REC-cue (Fig. 3a) displayed a distribution similar to that previously reported in humans expressing uncertainty^7^ and in animals when offered the possibility of escape^5^,^34^, restarting a trial^4^ or estimating confidence in a previous decision^6^,^14^,^35^,^36^. Other studies have reported the ability of apes and rhesus monkeys to search for more information when facing environmental uncertainty or self-doubt situations during a seeking information task^18^,^19^. Together these observations thus strongly support the notion that checking behavior relies on an assessment of decision confidence.

Rhesus monkeys have also been found capable of responding to, or abandoning, an uncertainty-monitoring task using a so-called deferred-feedback approach^37^. Interestingly, in such conditions, a clear dissociation is found between the cues of reinforcement-association and self-judgment of decision-making under uncertainty. This could in turn explain the asymmetrical degrees of success and checking we observed between “On” and “Off” status for a same ambiguity level. In our study, checking was mostly associated with a modification of a previous choice (i.e: reversing the REC-cue status; Supplementary Fig. 1), which generally led to an enhancement of the monkey’s performance. As in humans, therefore, monkeys use checking behavior as an adaptive function when facing doubt, allowing them to reassess their decision in order ultimately to improve goal-directed accuracy.

During the course of our experiments, we observed day-to-day fluctuations in the rate of checking behavior (Fig. 3c) that cannot be explained either by our experimental design or by any changes in the task protocol, which was rigorously maintained constant during daily data collection. Checking behavior triggered by uncertainty has been related to levels of anxiety in healthy subjects^7^ as well as in sub-clinical^38^,^39^ and clinical patients^7^,^40^. Indeed, its exacerbation is well known to be a central feature of OCD, an anxiety disorder that affects 2–3% of the population^1^,^2^. In order to investigate the impact of a monkey’s anxiety state on its checking behavior in our Check-or-Go paradigm, we measured salivary cortisol levels. This steroid hormone has been reported as a biological marker of stress and anxiety-state in both NHP^41^,^42^,^43^ and humans^44^,^45^,^46^, and its measurement in saliva offers the considerable advantage of being reliable and stress-free for the subject^41^,^42^,^47^,^48^.

Our analysis revealed that a monkey’s daily checking rate was positively correlated with levels of salivary cortisol present immediately prior to each session (Fig. 3d). This finding is therefore consistent with the conclusion that, as in humans^7^,^38^,^39^,^40^, a higher anxiety state leads monkeys to check more often, suggesting a contribution of anxiety to decision-making under uncertainty. This cortisol level association with checking behavior in our experiments could not be explained by an indirect impact of anxiety state on the animals’ behavior, since none of the behavioral parameters potentially indicating a global increase in motor activity were correlated with cortisol level (Supplementary Fig. 2a–c). Furthermore, we found an inverse correlation between salivary cortisol content and RT during the Confidence-Report step, indicating an impact of the anxiety state on the decision to check or not, favoring the adoption of a more cautious strategy when the animal is more anxious (Supplementary Fig. 2d). However, whether the anxiety factor affects the emergence of doubt through intolerance to uncertainty, the misrepresentation of the negative consequences of a decision (reward acquisition failure or punishment), or is more directly related to the checking behavior itself, requires further exploration^49^.

Finally, to identify the electrophysiological correlates of doubt-related checking behavior, we performed EEG recordings in cortical frontal areas (Fig. 4a). We first looked at the impact of REC-cue ambiguity on frontal event-related potentials (ERP) associated with decision-making processes. As reported in humans^50^,^51^,^52^, the ERP components N2 and P300, associated respectively with conflict and stimulus discrimination, were affected by ambiguity detection. Interestingly, the impact of ambiguity detection was opposite depending on the “On” or “Off” REC-cue status. Specifically, statistical analysis revealed that when the REC-cue was “On”, the N2 potential was smaller during High ambiguity trials for both monkey G (Fig. 4b and Supplementary Fig. 3a) and F (Supplementary Fig. 3a) at the AF_Z_ electrode (see Supplementary Fig. 4 for individual electrode data). On the other hand, an opposite effect was observed when the REC-cue was “Off”, with a larger N2 potential being recorded during High ambiguity trials for both the G (Fig. 4d and Supplementary Fig. 3b) and F monkeys (Supplementary Fig. 3b) at the AF_7_ electrode (see Supplementary Fig. 5 for individual electrode data).

In accordance with the implication of the N2 ERP in conflict detection, Szmalec *et al*. also observed a larger N2 potential associated with difficult discriminatory trials during an auditory discrimination task^50^. However, it is generally considered that the fronto-central N2 component has multiple functional correlates and could be sensitive, among others, to cognitive control or attentional processes^53^; This in turn could explain the observed effect of the “On” REC-cue ambiguity on N2 magnitude. Furthermore, these authors reported that increasing the predictive value of a stimulus led to an increase in N2 amplitude recorded at frontocentral sites^53^. It is therefore possible that a low ambiguity “On” REC-cue as well as a High ambiguity “Off” REC-cue, which are respectively associated with the maximal certainty of obtaining or failing to gain a reward, evoked similar peak levels of N2 activity. Nonetheless, our observations on the influence of ambiguity on P300 amplitude were in accordance with those of Johnson and Donchin^52^, since we found that the P300 mean potential was significantly larger as the stimulus ambiguity decreased (i.e., as discriminability increased), for both monkey G (Fig. 4b and Supplementary Fig. 3a,b) and monkey F (Supplementary Fig. 3a,b) at the AF_Z_ electrode (see Supplementary Fig. 4–5 for individual electrode data).

We then assessed the modulation of frontal neural activity related to the preparation of checking behavior. The N-40 component, identified as an electrophysiological marker of response selection in humans^54^, was significantly increased before checking initiation for both monkey G (Fig. 4c and Supplementary Fig. 3c) and F (Supplementary Fig. 3c) at the fronto-central (FC_1_) electrode (see Supplementary Fig. 6 for individual electrode data). In accordance with previous studies conducted in humans^7^,^55^,^56^,^57^,^58^, therefore, these electrophysiological data confirm the involvement of cortical frontal areas in the physiology of doubt and subsequent checking behavior in monkeys.

In conclusion, our study provides a novel animal behavioral model of checking behavior directly comparable to voluntary checking in humans^8^,^15^ and to the anxiety-induced checking observed in OCD patients^32^,^40^. Indirect behavioral measures have already allowed assessment of confidence in animals^8^,^9^,^13^, and doubt-based checking has also been previously reported in non-humans^18^,^19^. Nevertheless, these latter studies were based on information-seeking tasks allowing the animal to obtain more information when needed, but without offering the possibility to question a previously performed action. A more recent study reported an elegant experimental “checking paradigm” in which monkeys were offered the possibility of checking for potential additional reward delivery^59^. The originality of this task was its involvement in a distinct form of checking that was most probably triggered by motivation and curiosity. In contrast, the paradigm we used provided animals with an opportunity to check a previously carried-out action in order to readjust their decision process.

Our results confirm that, as in humans, checking behavior in NHP is an adaptive behavior that enables accuracy improvement, fluctuates with anxiety, and is associated with activity in frontal brain areas. In an evolutionary perspective, therefore, we propose that human checking compulsion derives and escalates from this fundamental motivational behavior (“something is wrong… I need to check”^3^,^60^) that contributes to relieving anxiety caused by doubt and uncertainty^38^,^40^. Hence, this animal model should help not only in clarifying the biological basis of psychiatric diseases such as OCD^61^, but also to decipher more general characteristics of decision-making processes^9^.

## Materials and Methods

### Ethics statement

Veterinarians skilled in NHP maintenance supervised animal care in strict accordance with the European Community Council Directive for animal experimental procedures. Experiments were conducted in accordance with the Council Directive 2010 (2010/63/UE) of the European Community and the National Institute of Health Guide for the Care and Use of Laboratory Animals. The protocol used was validated by the Ethical Committee for Animal Research CE50 (Agreement number: 5012054-A).

### Subjects

All experiments were conducted on two male rhesus macaques (*Macaca mulatta*; monkeys G and F) that were 5 and 6 years old, respectively, at the beginning of data collection. They were housed in individual primate cages with free access to food and water, except the day before experimentation during which they were restrained from drinking. Animals were weighed weekly and allowed to gain weight normally.

### Experimental paradigm

**Figure 1:**
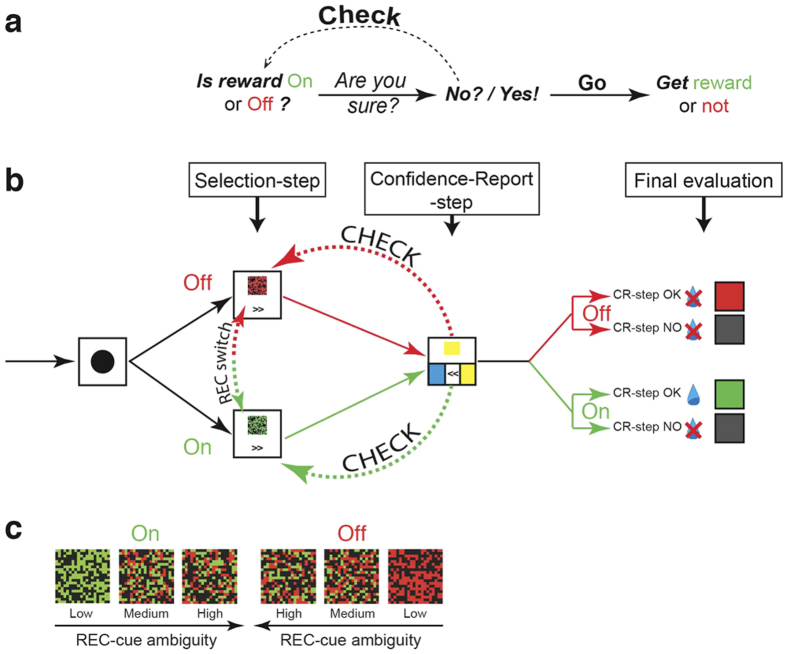
The Check-or-Go Task. (**a**) Schematic of Check-or-Go behavior. (**b**) Check-or-Go task experimental paradigm. After a warning stimulus, reward availability was indicated by a randomly-chosen REC-cue. During this initial Selection-step, the monkey could either switch the reward status (from “On” to “Off” or “Off” to “On”) by touching the REC-cue or advance to the next step by pressing the “next-step” arrow (>>). During the following Confidence-Report-step, the animal could either decide to move to the final evaluation by answering the color-matching question or to check reward availability at the Selection-step by pressing the “Go back-arrow” (<<). The monkey could check (and reverse) the reward status indefinitely. During the evaluation period, both reward and visual feedback were provided according to the reward status and success in the color-matching step. (**c**) Examples of REC-cues depicted for Low, Medium and High ambiguity levels for each reward status (“On” = reward available, “Off” = reward unavailable). Note that the opposing directions of the two arrows correspond to increasing levels of ambiguity.

We designed an experimental protocol that allows the animal to check repetitively and change the availability of a reward before taking the final decision towards actually obtaining the reward (Fig. 1a). The Check-or-Go task was divided into two main steps (Fig. 1 and see Supplementary Videos 1, 2):

(1) A Selection-step during which the availability of the reward could be altered.

(2) A Confidence-Report-step during which the monkey could confirm acceptance of the first step or report a no-confidence decision by selecting a go-back function.

Each trial began with a warning stimulus in the form of an on-screen black circle (diam. 5 cm), during which the monkey was instructed to remain stationary while holding the control handle. After a 200–500 ms delay, a switchable REward Control cue (REC-cue) that conditioned the reward status at the end of the trial appeared in the upper part of the monitor simultaneously with a “next-step” arrow (>>) at the bottom of the screen (Fig. 1b). A predominantly green REC-cue indicated that the reward was available at the end of the trial (reward status “On”), whereas a predominantly red REC-cue meant that the reward was unavailable at the end of the trial (reward status “Off”). The REC-cue consisted of an ambiguous squared visual cue (20 × 20 pixels) consisting of 200 black task-irrelevant squares and a total of 200 colored (green and/or red) squares randomly positioned and combined in different proportions in order to create three distinct levels of visual discrimination, i.e., REC-cues with low, medium and high-ambiguity (see examples in Fig. 1c). During this first step, the animal could either reverse the reward status by pressing the REC-cue itself which resulted in a switch from one color (and thus reward status) to the other, or continue with the same reward status by pressing the “next-step” arrow, or wait for a further 2000 ms. After an additional 500–600 ms delay, the second stage of the task, named the Confidence-Report-step, began with either a blue or yellow unambiguous colored cue in the upper part of the monitor, as well as two colored square (yellow and blue) targets. Crucially, a “Go back-arrow” (<<) flanked by the two colored targets was presented simultaneously. During this second step, animals could either choose to go on to the trial’s final evaluation step by answering the unambiguous color-matching test, or to check the reward status by pressing the “Go back-arrow” which led back to the initial Selection-step. The monkey was able to check (and reverse) the reward status for as many times as it wished. To reach the trial’s evaluation phase, the animal had to perform a color-matching task by pressing a colored target at the bottom of the screen. After a further 200–500 ms delay, the evaluation period started with the display of a colored visual feedback signal accompanied, or not, by the delivery of a drop of juice as a reward. Four different trial results could be obtained (Fig. 1b, far right side) depending on the reward status at the end of the trial and the animal’s performance in the color-matching task. In order to effectively gain the reward, the two following conditions required fulfilling: the REC-cue had to be green (“On” reward status) and the color-matching task had to be performed correctly. Hence only trials ending with the reward status “On” and a correct color matching were rewarded by a drop of juice during display of a green feedback screen for 500–800 ms. Trials ending with the reward status “Off” (i.e. reward unavailable) despite a correct color-matching resulted in no reward delivery and a 5–15 s time-out penalty period during which a red feedback screen was displayed. The few trials in which an error was made at the color matching stage also resulted in no reward delivery and were accompanied by a black feedback screen display for either 500–800 ms or 5–15 s, respectively according to the “On” or “Off” reward status.

### Behavioral training

Behavioral training took approximately 10 months. Animals were trained for at least one hour per session, 5 times a week. During each training session, the animal was seated in a primate chair within arm’s reach of a tangent infrared-screen (IR Touch, China) coupled to a 15″ flat screen monitor. The left arm was restrained so that the monkey could touch the screen with its right hand only. Animals were trained to keep their right hand on a hand-rest that contained a position sensor (the “handle”) that enabled the accurate detection of both beginning and ending of movements. A custom Labview program (National Instrument, USA) controlled the presentation of visual stimuli as well as reward delivery and monitored the touch-screen and the handle. Reward (fruit juice or water) was delivered via a solenoid and a liquid reward pipe attached to the monkey’s chair.

At the beginning of experimental data collection, the animal was already trained to perform a color-matching task with several color combinations and ambiguous visual cue discriminability, and was familiar with the REC-cue color-reward association of green and red corresponding to reward availability and unavailability, respectively. Data collection started once the animals had obtained and stabilized their success rates for the three levels of difficulty in the visual-cue discrimination: near 100% of correct trials for the lowest ambiguity REC-cue, ~80% for the intermediate ambiguity REC-cue and ~60% for the highest ambiguity REC-cue. During data collection, the proportions of green and red in the REC-cue were monitored in order to adjust visual discrimination difficulty and maintain the success rate at ~100%, 80% and 60% for the lowest, intermediate and highest ambiguity REC-cues, respectively.

### Surgical procedures

**Figure 4:**
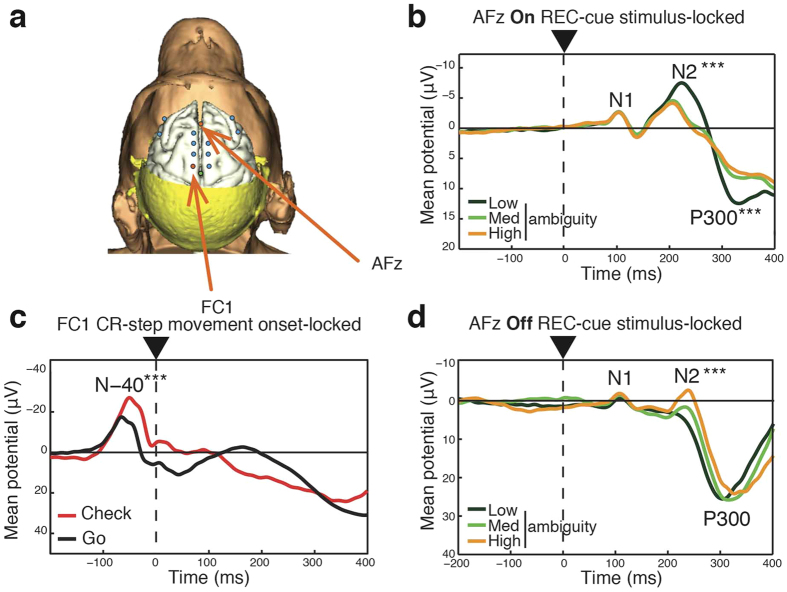
Frontal EEG during the Check-or-Go task. (**a**) EEG recording electrode location. (**b,c,d**) Grand average ERP from 46 sessions with monkey G. ***P < 0.001 for *Bonferroni’s post hoc* test. See also Supplementary Fig. S3 for detailed statistics. (**b**) N2 and P300 components at the AFz electrode were modulated by REC-cue ambiguity when an “On” REC-cue (i.e. predominantly green) was displayed at the onset of the selection-step. (**c**) The fronto-central N-40 component was significantly delayed and increased before checking initiation. (**d**) N2 components at the AF7 electrode were modulated by REC-cue ambiguity when an “Off” REC-cue (i.e. predominantly red) was displayed at the onset of the selection-step.

13 electrodes were implanted over the frontal dorso-medial and dorso-lateral monkey G and F areas (corresponding approximately to CPz, C1, C2, FC1, FC2, F1, F2, AF3 AF4, FPz, F3, F5, F4, F6 sites in humans; see Fig. 4a for exact electrode locations).

Surgical procedures were conducted under aseptic conditions and under general anesthesia (ketamine hydrocloride (10 mg/kg) following atropine sulfate (0.05 mg/kg) and diazepam (0.5 mg/kg) exposure in preparation for surgery and isoflurane (1.5–2%) during actual surgery). Antibiotic (amoxyciline, 15 mg/kg) and analgesic (ketoprofen, 2 mg/kg) treatments were given for 1 week after surgery. EEG electrode positioning was performed under MRI-guided surgery (Brainsight, Rogue Research, Canada). Neuroimaging was performed on a Siemens 3T MAGNETOM Trio TIM using 0.5 mm voxels. EEG electrode design was based on previously published procedures^62^; the electrode implants were constructed from 8.5 cm Teflon-coated braided stainless steel wire and solid gold amphenol pins (Cooner Wire, USA; A-M System, USA). One end of each wire was connected to an electrode interface EIB-16 board EIB-16 (Neuralynx, USA), while the crimped gold pin on the other end was ground down to ~1 mm in diameter. Holes of similar diameter were drilled into the surface of the skull (~3 mm thick), allowing the gold terminal end of the electrode to be tightly inserted and then covered with a small amount of acrylic cement. When all the EEG electrodes had been implanted, connector ends were placed into a plastic chamber for protection and to allow access during recording sessions. The electrode leads that were not embedded in acrylic were covered by skin that was sutured back over the skull. This allowed for the EEG electrodes to be minimally invasive once implanted.

### EEG recordings

Electrical potential changes were recorded with a multichannel data acquisition system (Alpha Omega, Israel) with a preamplifier band pass of 1–200 Hz and digitized at 5,000 Hz. EEG data were collected during 46 and 24 recording sessions for monkey G and F, respectively. A baseline epoch was defined as the potential recorded at −500 to −100 ms before the beginning of an event (REC-cue onset for N1, N2 and P300 analyses, and movement onset during the Confidence-Report-step for N-40 analysis). Peak magnitudes for the four ERP were then computed for each session using a peak detection software tool and expected ambiguity and response effects were tested using one-way ANOVA followed by *Bonferroni’s post hoc* test when the main effect was significant at *P* < 0.05.

### Salivary cortisol dosage

During one month, individual saliva samples were collected using an infant swab (Salimetrics, USA) immediately before each experimental session. Based on the literature^47^,^48^, we trained the monkeys to chew the swab for about 80 s and then spit it out into a sterile kidney dish. Immediately after collection, the swab was transferred into a swab storage tube (Salimetrics, USA) and centrifuged for 15 min at 3500 rpm. The extracted saliva was then stored at −80 °C until assayed. Samples were analyzed in duplicate using an enzyme immuno-assay for salivary cortisol following manufacturer’s instructions (Salimetrics, USA). The cortisol data were standardized [X _standardized_ = (X-mean)/standard deviation] in order to pool data from both animals.

### Statistical analysis

Data collection and analyses took place over 106 and 136 sessions, corresponding to a total of 50621 and 65995 trials for monkey G and F, respectively. For the sake of clarity and space, results are mostly shown for pooled data, although for a given analysis, we first assessed for statistically significance for each monkey separately. Behavioral analyses were performed using custom-written Matlab scripts and ERP analyses using EEG-lab and custom-written Matlab scripts. The very few trials (0.57%) in which an error was made in color matching (during the Confidence-Report-step) were excluded from the analyses. Data are expressed as mean ± s.e.m. Behavioral choices (performance and checking rate) are expressed as percent of trials. Mean differences between reaction times (RT) were determined using either a one- or two-way analysis of variance (ANOVA) followed by *Bonferroni’s post hoc* test when the main effect was significant at *P* < 0.05. For performance and checking rates, we used two-sided *Chi*-*square* tests for all comparison between trial type and before-after checking. The correlation between salivary cortisol and checking rate was assessed using the *Pearson* correlation test. The alpha level was set at 0.05 for every statistical test. No statistical methods were used to predetermine sample sizes, but these were similar to sample sizes routinely used in the field for similar experiment^30^.

## Additional Information

**How to cite this article**: Bosc, M. *et al*. Checking behavior in rhesus monkeys is related to anxiety and frontal activity. *Sci. Rep.* **7**, 45267; doi: 10.1038/srep45267 (2017).

**Publisher's note:** Springer Nature remains neutral with regard to jurisdictional claims in published maps and institutional affiliations.

## Supplementary Material

Supplementary Information

Supplementary Video 1

Supplementary Video 2

## Figures and Tables

**Figure 2:**
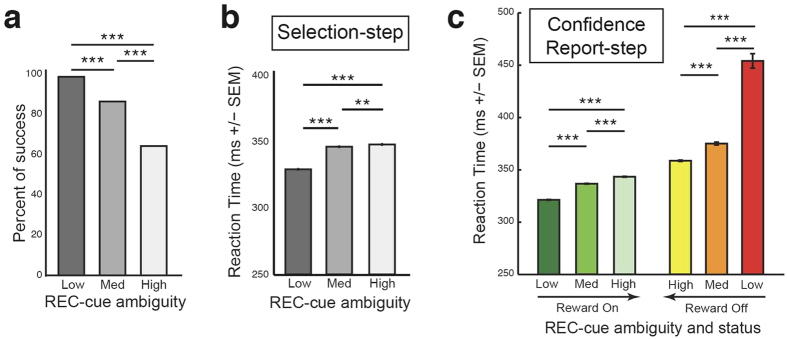
Impact of REC-cue ambiguity on behavioral confidence. (**a**) The rate of task success decreased significantly as the REC-cue ambiguity level increased (*Chi2*-test, ***P < 0.001). (**b**) Reaction time (RT) increased significantly with the REC-cue ambiguity level during the Selection-step (***P < 0.001; **P < 0.01 for *Bonferroni’s post hoc* test). (**c**) RTs during the Confidence-Report-step were significantly dependent on REC-cue ambiguity level, and were oppositely modulated according to “On”/“Off” reward status (***P < 0.001 for *Bonferroni’s post hoc* test). All RT values in (**b,c)** are means ± SEM. Note that as in Fig. 1c the level of ambiguity is displayed in an inverse manner for “On” and “Off” REC-cue status as indicated by arrows.

**Figure 3:**
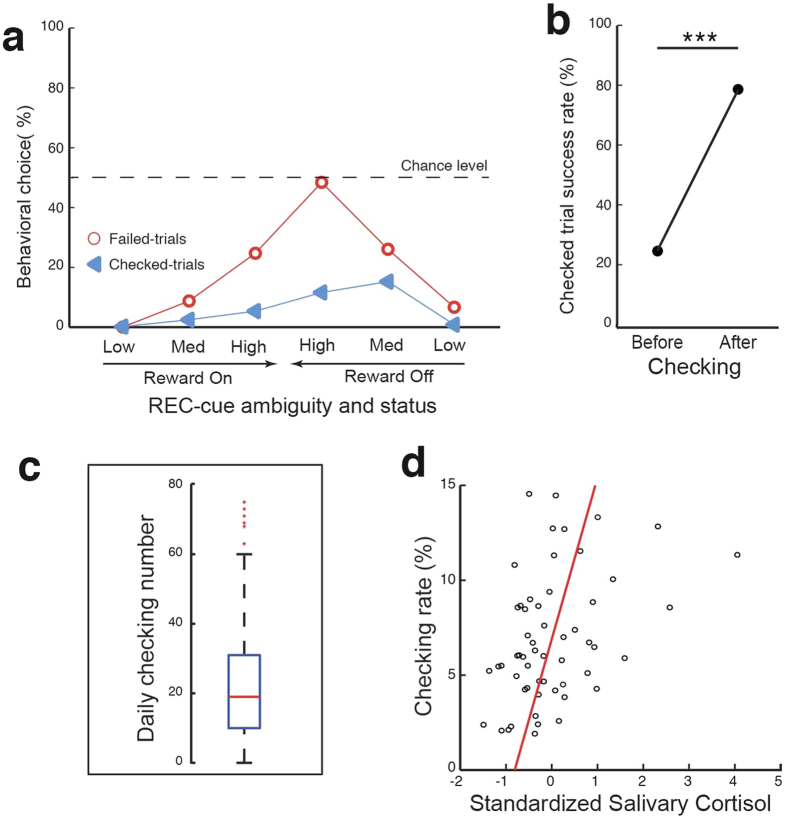
Checking behavior and anxiety during the Check-or-Go task. (**a**) Rates of failed- and checked-trials as a function of REC-cue ambiguity and reward status (*n* = 242 sessions). (**b**) Percentage success of checked trials before and after checking. Performance was significantly improved after checking (*Chi2*-test, *P* < 0.001). (**c**) Boxplot of checking number over 242 sessions. Box limits represent quartiles (25%, 75%) and the median is indicated by a red line. Whiskers show a range of up to 1.5 times the interquartile range; red crosses indicate outliers. (**d**) Checking rate as a function of salivary cortisol levels measured at the beginning of each session (*n* = 56 sessions; *Pearson* correlation: *r* = 0.4; *P* < 0.01).
