# Supplementary material for: Checking behavior in rhesus monkeys is related to anxiety and frontal activity
Source: Sci Rep. 2017 Mar 28;7:45267. doi: 10.1038/srep45267 (PMC5368664; doi:10.1038/srep45267)
Supplement: Supplementary Information [file srep45267-s1.pdf]

Supplementary Information

Checking behavior in rhesus monkeys is related to anxiety and frontal activity

Marion Bosc[1,2], Bernard Bioulac[1,2,3], Nicolas Langbour[4], Tho-Hai Nguyen[1,2], Michel Goillandeau[1,2], Benjamin Dehay[1,2], Pierre Burbaud[1,2,3], Thomas Michelet*[1,2,5]

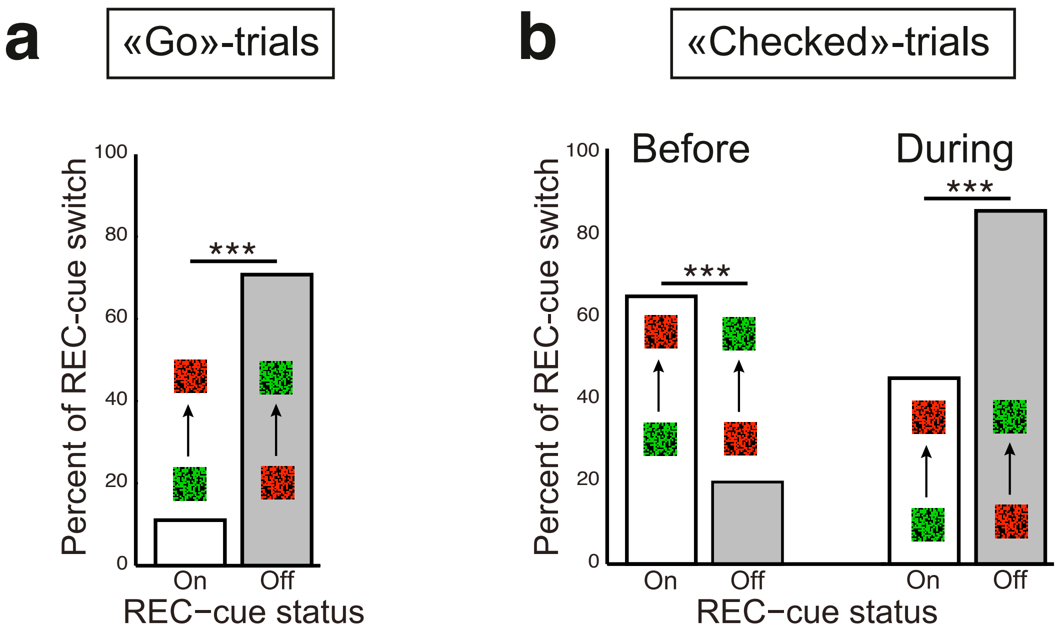

Supplementary Figure 1. Dependence of REC-cue switching behavior on REC-cue status.

Rates of REC-cue switching during the Selection-step varied significantly with reward status (a) during "Go"-trials ($X^2_{1,n=117161}$= 43241.0, P<.001), and (b) before ($X^2_{1,n=5095}$= 1075.7, P<.001) and during ($X^2_{1,n=5152}$= 916.1, P<.001) actual checking in "Checked"-trials. Note that monkeys switched the REC-cue significantly more when the reward was not available ("Off") compared to when it was available ("On") during "Go"-trials and during checking. However, they switched the REC-cue significantly more when the reward was available ("On") compared to when it was not available ("Off") before a checking.

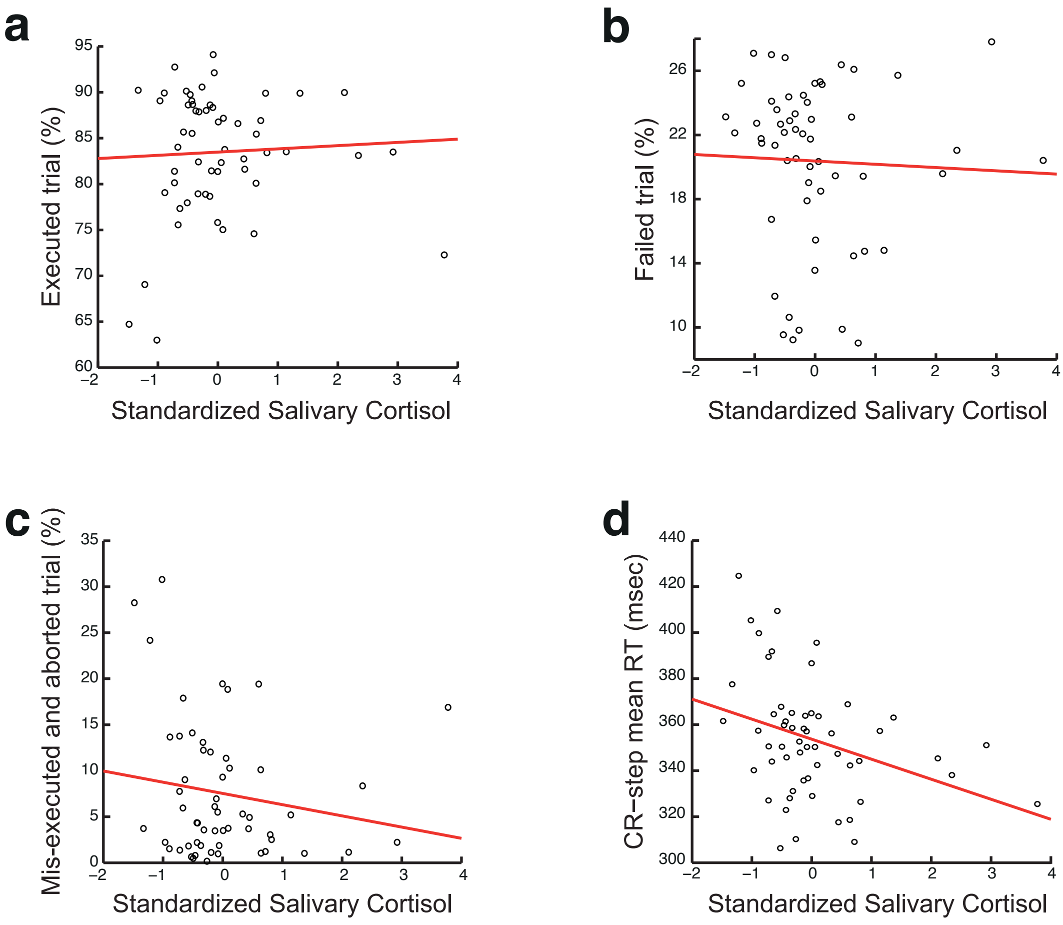

Supplementary Figure 2. Salivary cortisol and behavior.

(a) Percentages of successfully executed trials as a function of salivary cortisol levels measured at the beginning of each session (n=56 sessions; *Pearson* correlation: r= 0.05; P=NS). (b) Percentages of failed trials vs salivary cortisol levels at the beginning of each session (n=56 sessions; *Pearson* correlation: r= -0.04; P=NS). (c) Percentages of attentional error trials (aborted) vs salivary cortisol levels at the beginning of each session (n=56 sessions; *Pearson* correlation: r= -0.16; P=NS). (d) Mean RT during the Confidence-Report step vs salivary cortisol levels at the beginning of each session (n=56 sessions; *Pearson* correlation: r= -0.34; P= 0.01).

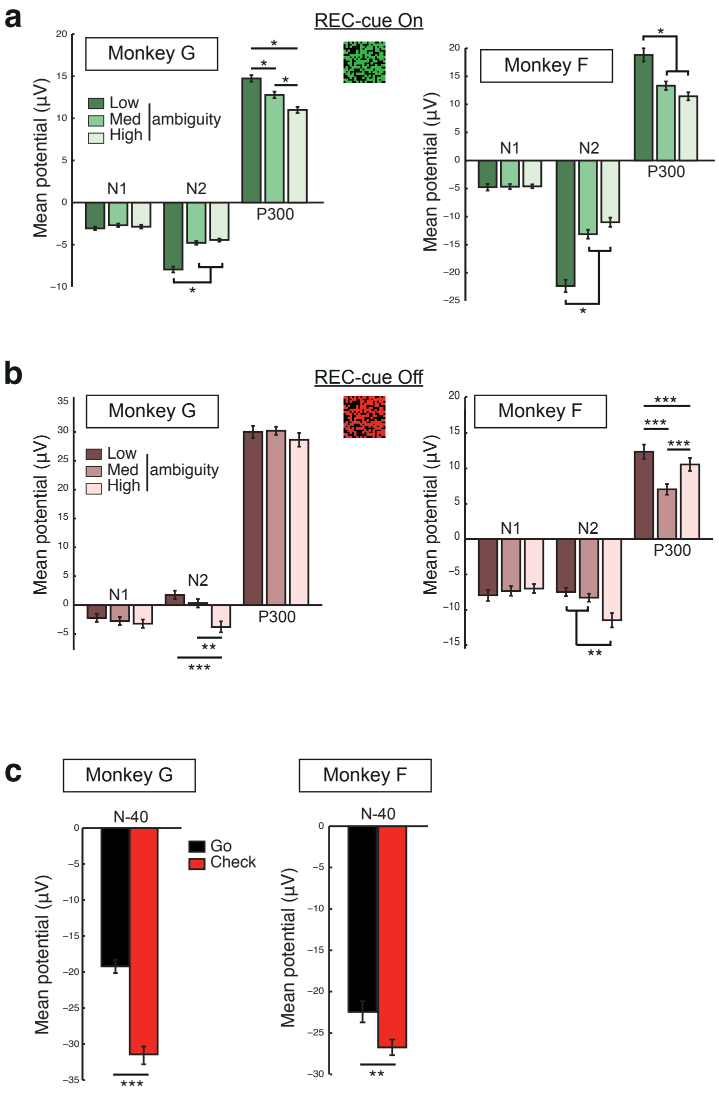

Supplementary Figure 3. N1, N2, P300 and N-40 component magnitudes during the Check-or-Go task.

(a) When an "On" (i.e. green) REC-cue was displayed during the Selection-step, N2 and P300 magnitudes at the AFz electrode decreased as REC-cue ambiguity increased for both monkey G (N2: $F_{2,138}$= 54.7, P<.001; P300: *F*2,135= 24.3, *P*<.001) and F (N2: $F_{2,72}$= 43.5, *P*<.001; P300: $F_{2,72}$= 17.7, *P*<.001), while the N1 ERP component was not affected. * *P*< 0.05 for *Bonferroni's post hoc* test. (b) Conversely, when an "Off" (i.e. red) REC-cue was displayed during the Selection-step, N2 magnitude at the AF7 electrode increased as REC-cue ambiguity increased for both monkey G ($F_{2,135}$= 12.2, P<.001) and F ($F_{2,69}$= 7.9, *P*<.001). Note that when the REC-cue was "Off", the P300 ERP component magnitude was also modulated differently according to the three levels of ambiguity for monkey F ($F_{2,66}$= 9.3, *P*<.001), while N1 was not affected for both monkeys. *** *P*<

0.001 for *Bonferroni's post hoc* test. (c) The N-40 component at the fronto-central electrode was significantly increased before checking initiation for both monkey G ($F_{1,41}$= 88.5, P<.001) and F ($F_{1,21}$= 8.9, P<.001). ** P< 0.01; *** P< 0.001 for *Bonferroni's post hoc* test. All values are means ± SEM.

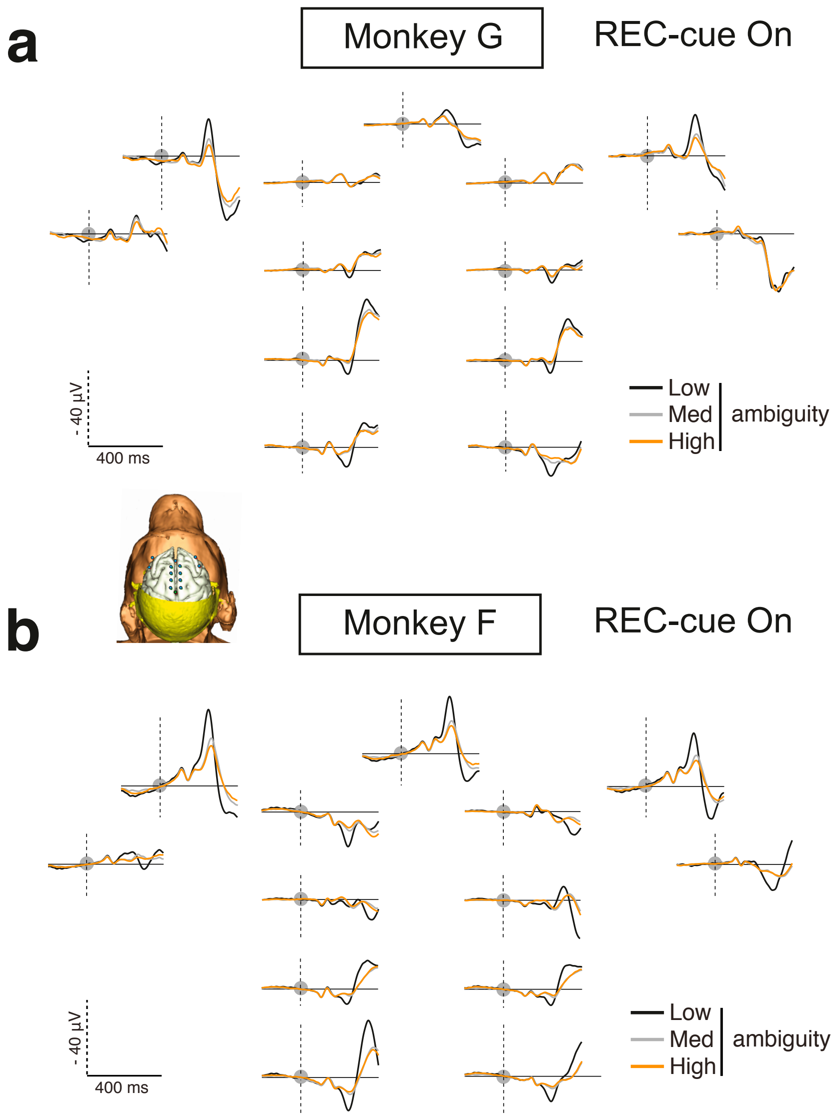

Supplementary Figure 4. Frontal EEG signals at the onset of the Selection-step during trials with an "On" REC-cue.

(a,b) Grand average ERPs recorded at individual electrodes at the onset of the Selection-step during trials starting with an "On" (green) REC-cue for both monkey G (a) and F (b), and with Low (black), Medium (Grey) and High (Orange) ambiguity levels.

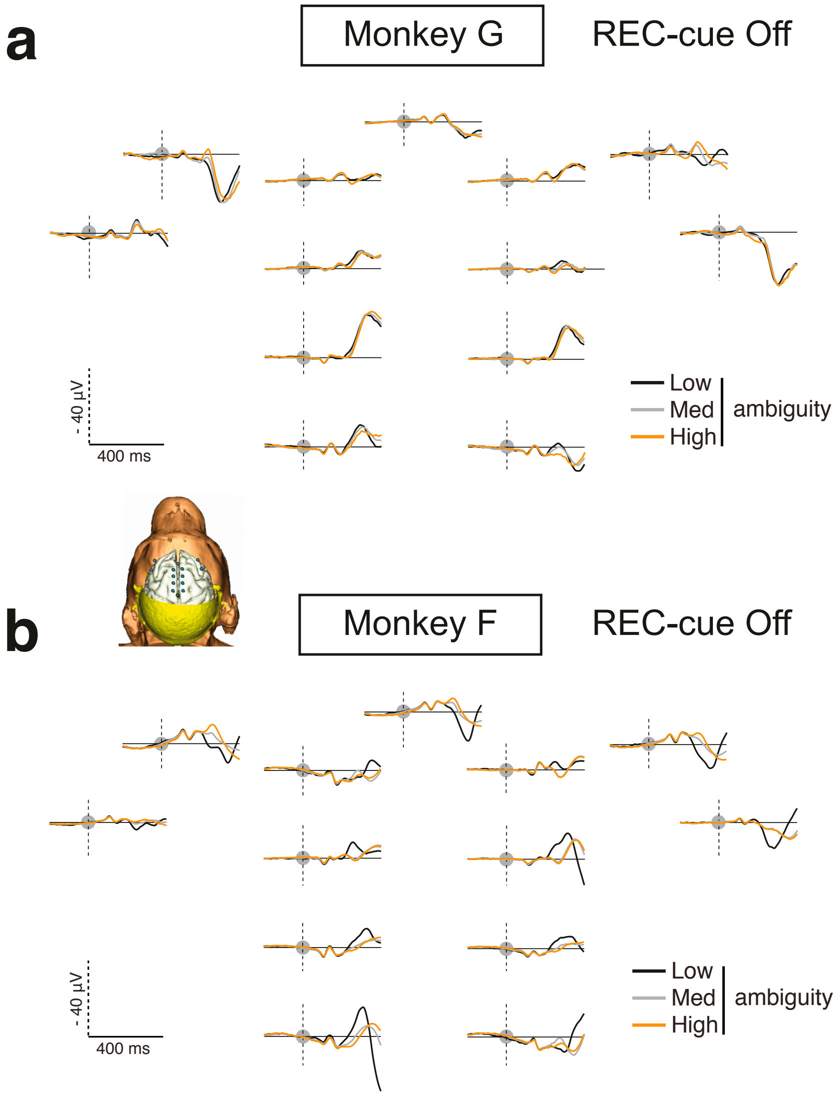

Supplementary Figure 5. Frontal EEG signals at the onset of the Selection-step during trials with an "Off" REC-cue.

(a,b) Grand average ERPs at individual electrodes at the onset of the Selection-step during trials starting with an "Off" (red) REC-cue for monkey G (a) and F (b), and with Low (black), Medium (Grey) and High (Orange) ambiguity levels.

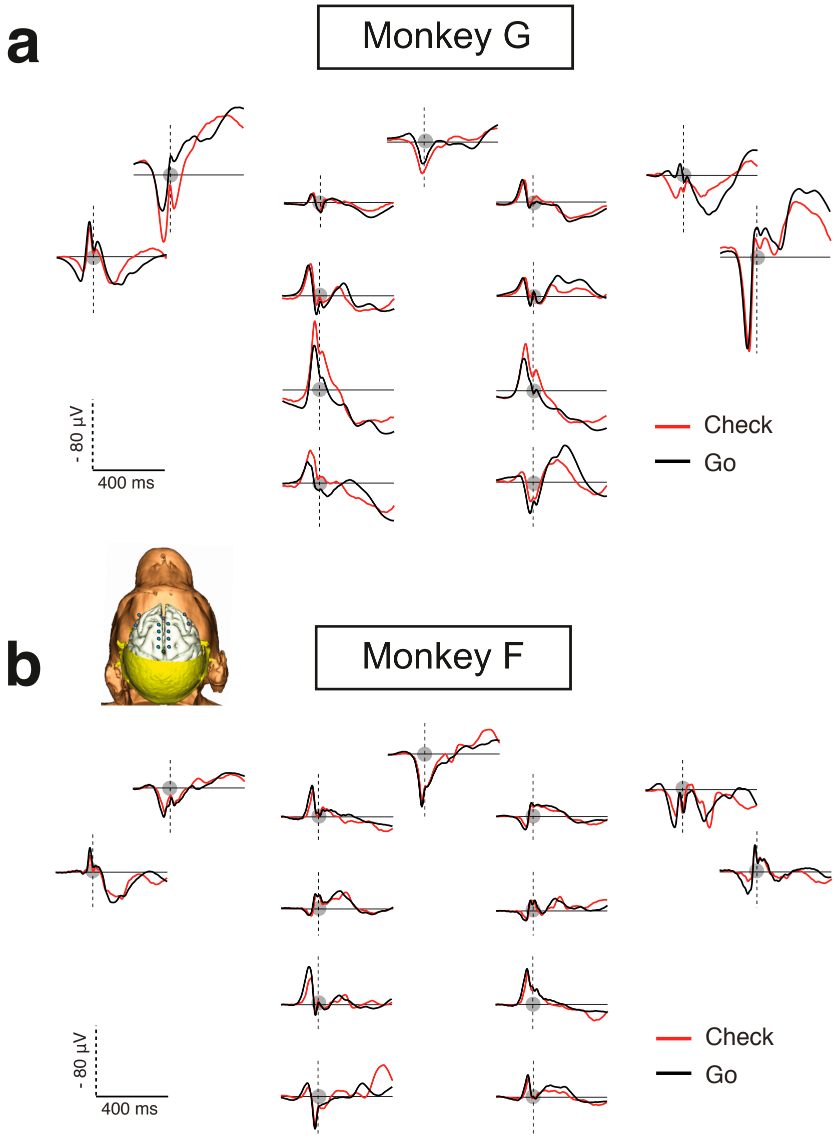

Supplementary Figure 6. Frontal EEG signal at the onset of the movement during Confidence-Report-step.

(a,b) Grand average ERPs at each electrode at the onset of movement during the Confidence-Report-step for monkey G (a) and F (b), for trials during which the animal decided to check (red) or confirm its initial choice (black).

Supplementary Video 1. Examples of “Go”-trials.

The video shows two filmed trials of the Check-or-Go task, without checking behavior (“Go”-trials). The first example is a trial successfully performed by the monkey as the reward status is “On” at the end of the trial, whereas the second example depicts a failed trial since the reward status is “Off” at the end of the trial. Each example is first played at normal speed and then in slow motion (x0.5).

Video S2. Examples of “Checked”-trial.

The video shows two filmed trials of the Check-or-Go task, with checking behavior (“Checked”-trials). During the first example, the monkey performs checking on an “On” REC-cue and then decides not to change it, whereas in the second example, the monkey conducts checking of an “Off” REC-cue then switches its status from “Off” to “On”. For both examples the reward status is “On” at the end of the trial and the monkey obtains a reward. Each example is first played at normal speed and then in slow motion (x0.5).
